# Deep brain stimulation for neurological disorders: a protocol for a systematic review with meta-analysis and Trial Sequential Analysis of randomised clinical trials

**DOI:** 10.1186/s13643-022-02095-z

**Published:** 2022-10-13

**Authors:** Johanne Juul Petersen, Sophie Juul, Caroline Kamp Jørgensen, Christian Gluud, Janus Christian Jakobsen

**Affiliations:** 1 Copenhagen Trial Unit, Centre for Clinical Intervention Research, The Capital Region, Copenhagen University Hospital – Rigshospitalet, Blegdamsvej 9, Copenhagen Ø, 2100 Copenhagen, Denmark; 2 Department of Regional Health Research, Faculty of Health Sciences, University of Southern Denmark, J.B. Winsløws Vej 19, 3, Odense C, 5000 Odense, Denmark

**Keywords:** Deep brain stimulation, Parkinson’s disease, Essential tremor, Dystonia, Epilepsy, Beneficial effects, Adverse effects

## Abstract

**Background:**

Deep brain stimulation has been used since the 1980s for neurological disorders and the USA and Europe have now approved it for Parkinson’s disease, essential tremor, dystonia, and epilepsy. Previous reviews have assessed the effects of deep brain stimulation on different neurological disorders. These reviews all had methodological limitations.

**Methods:**

This is a protocol for a systematic review based on searches of major medical databases (e.g. MEDLINE, EMBASE, CENTRAL) and clinical trial registries. Two review authors will independently extract data and conduct risk of bias assessment. We will include published and unpublished randomised clinical trial comparing deep brain stimulation versus no intervention, usual care, sham stimulation, medical treatment, or resective surgery for Parkinson’s disease, essential tremor, dystonia, or epilepsy. The effects of deep brain stimulation will be analysed separately for each of the different diagnoses. Primary outcomes will be all-cause mortality, disease-specific symptoms, and serious adverse events. Secondary outcomes will be quality of life, depressive symptoms, executive functioning, level of functioning, and non-serious adverse events. Data will be analysed using fixed-effect and random-effects meta-analyses and Trial Sequential Analysis. Risk of bias will be assessed with the Cochrane Risk of Bias tool—version 2, an eight-step procedure to assess if the thresholds for clinical significance are crossed, and the certainty of the evidence will be assessed by Grading of Recommendations, Assessment, Development and Evaluations (GRADE).

**Discussion:**

Deep brain stimulation is increasingly being used for different neurological diseases, and the effects are unclear based on previous evidence. There is a need for a comprehensive systematic review of the current evidence. This review will provide the necessary background for weighing the benefits against the harms when assessing deep brain stimulation as intervention for individual neurological disorders.

**Systematic review registration:**

PROSPERO 306,556.

**Supplementary Information:**

The online version contains supplementary material available at 10.1186/s13643-022-02095-z.

## Background

### Description of the conditions

Neurological disorders are the second leading cause of death worldwide and encompass a wide range of conditions affecting the brain [1]. Among these, certain movement disorders and epilepsy are some of the more recognised [1]. Movement disorders cover several different neurological conditions including Parkinson’s disease, essential tremor, and dystonia [2].

Parkinson’s disease is estimated to affect 1% of the population above 55 years with a rising incidence and prevalence [1, 3]. Parkinson’s disease has a complex pathophysiology with neuronal death particularly affecting the dopaminergic neurons, which affects neurological pathways through the basal ganglia resulting in bradykinesia, tremor, and rigidity [4, 5]. The symptoms of Parkinson’s disease are, however, diverse and may include motor signs related to non-dopaminergic transmission and non-motor features as well [5]. Parkinson’s disease is commonly treated pharmacologically with levodopa, but many patients become resistant to levodopa over time, and levodopa has several adverse effects [4].

Essential tremor is estimated to affect 1% of the population worldwide [6]. Essential tremor typically affects the upper extremities with hyperkinetic tremor, but it may spread to additional parts of the body [7]. The precise pathophysiology behind essential tremor is controversial but probably involves pathological rhythmic oscillation [7]. Essential tremor is usually treated pharmacologically with propranolol and primidone [7]. While the pharmacological treatment may result in a 70% reduction in tremor, about 50% of the patients have no effect of medications and propranolol and primidone are often associated with adverse effects [6, 7].

Dystonia has been estimated to affect 0.7% of the population above 50 years [8]. The disorder covers a group of hyperkinetic movement disorders characterised by involuntary, sustained, or repetitive muscle contraction, affecting one or more body regions [8, 9]. The pathophysiology behind dystonia is complex with involvement of the sensory system, overexcitability in the motor cortex, and alterations in the basal ganglia circuitry [8]. The usual treatment is botulinum toxin injection, surgical or pharmacologically with anticholinergics, baclofen, benzodiazepines, or levodopa [9, 10].

Epilepsy is a collection of neurological seizure disorders affecting 0.5 to 1% of the population [11, 12]. It is characterised by spontaneous and recurring seizures caused by an imbalance in the inhibitory and excitatory signalling leading to excessive and synchronous neuronal firing [11, 13]. Epilepsy is typically treated with anti-epileptic drugs such as valproic acid or carbamazepine or the large number of newer anti-epileptic drugs [11, 14]. However, 40% of the pharmacologically treated patients show drug-resistant epilepsy while many experience unacceptable adverse effects [13].

### Description of the intervention

Deep brain stimulation is a neurosurgical intervention for neurological and psychiatric disorders associated with pathophysiological neuronal circuits [15]. Deep brain stimulation has been used since the 1980s [16]. As intervention for neurological disorders, it is now approved in the USA and Europe for Parkinson’s disease, essential tremor, dystonia, and epilepsy [16].

Deep brain stimulation uses high frequency stimulation which theoretically has an inhibitory effect on the involved pathological neuronal circuits [16]. However, the precise mechanisms behind its effects are largely unknown [16]. Deep brain stimulation may work by disrupting or counteracting pathological neuronal pathways using electrical stimulation with constant current or voltage [7, 16]. The high frequency stimulation is thought to minimise pathological communication, possibly by jamming the involved area [7, 13]. It has also been proposed that the stimulation affects metabolic changes and long-term neuroplasticity [15, 16].

A pulse generator plus a battery placed underneath the collar bone provides the electrical stimulation and records the neuronal electrical activity [13, 16]. The pulse generator provides the stimulation through wires placed underneath the skin which are then connected to one or more electrodes implanted in prespecified brain areas [13, 15]. The placement of the electrodes and thereby the stimulation site is dependent on the neurological disorder treated [15].

In Parkinson’s disease, the usual placement of the electrodes is the subthalamic nucleus or the internal or external globus pallidus of the brain [15, 16]. In essential tremor, the usual placement is the ventral intermedius nucleus [16]. In dystonia, the usual placement is globus pallidus while also thalamus and the subthalamic nucleus have been used [16]. In epilepsy, the placement of the electrodes can be either in an area of the brain thought to have a pacemaker, triggering, or gating position in the epileptic network, or it can be in the ictal onset zone [17]. Hence, the placement of the electrodes in epilepsy is often in thalamus, hippocampus, or the ictal onset zone, if this has been identified [15, 16].

### Why is it important to do this review?

We have identified several previous reviews and meta-analyses assessing the effects of deep brain stimulation.

We identified five reviews assessing the effects of deep brain stimulation for Parkinson’s disease [18–22]. These reviews generally concluded that deep brain stimulation seemed to improve disease progression, severity, and quality of life, while there were some risks of adverse events [18–22]. Three of these reviews assessed deep brain stimulation for Parkinson’s disease comparing different stimulation sites [18, 20, 22], one review compared deep brain stimulation versus best medical treatment [19], and one review compared different stimulation sites and best medical treatment [21]. These reviews have been limited by not publishing a protocol before the literature search began [18–22], not searching all relevant databases [18–22], not employing trial sequential analysis methods to control random errors [18–22], not assessing adverse effects [18, 21, 22], and not assessing the certainty of evidence using GRADE [18, 19, 21, 22].

We identified two reviews assessing the effects of deep brain stimulation for essential tremor [23, 24]. These reviews generally concluded that deep brain stimulation seemed to improve symptoms, while there was some risk of adverse events [23, 24]. One of these reviews assessing deep brain stimulation for essential tremor compared efficacy in patients with different tremor characteristics [24]. This review included observational studies in addition to randomised clinical trials in their meta-analyses [24]. The other review compared deep brain stimulation versus lesion surgery [23]. This review pooled patients with various causes for the tremor in their meta-analyses [23]. Hence, no systematic review with only randomised clinical trials assessing deep brain stimulation for essential tremor has been conducted. These previous reviews have also been limited by not searching all relevant databases [23, 24], not employing Trial Sequential Analysis to control random errors [23, 24], not assessing adverse effects [23], and not assessing the certainty of evidence using GRADE [23, 24].

We identified one review assessing the effects of deep brain stimulation for dystonia [25]. This review concluded that deep brain stimulation seemed to improve symptoms, while the improvement of quality of life and the risk of adverse events were uncertain [25]. This review assessing deep brain stimulation for dystonia compared deep brain stimulation versus placebo, sham intervention, or best medical care [25].

We identified one review assessing the effects of deep brain stimulation for epilepsy [26]. This review concluded that deep brain stimulation seemed to reduce seizure frequency depending on the placement of the electrodes [26]. This review compared deep brain stimulation and cortical stimulation versus sham stimulation, resective surgery, medical treatment or other neurostimulation treatments [26]. This review is limited by not employing trial sequential analysis methods to control random errors [26].

The characteristics and results of these previous reviews are summarised in Table 1.

**Table 1:** Overview of previous reviews of studies of deep brain stimulation for neurological disorders

Authors	Year of publication	Only RCTs included	Information sources	No. of studies included	No. of patients	Published protocol	Assessment of benefits	Assessment of adverse effects	GRADE assessment	Assessment of risk of bias	TSA	Conclusion
*Parkinson’s disease*												
Wong et al [18]	2019	Yes	PubMed, Web of Science, CENTRAL, clinicaltrials.gov	5	489	No	Yes	No	No	Only publication bias	No	Deep brain stimulation is effective in tremor suppression regardless of target
Bratsos et al [19]	2018	Yes	EMBASE, MEDLINE, CENTRAL	8	1189	No	Yes	Yes	No	Yes	No	Deep brain stimulation is effective to improve impairment and quality of life but with a higher risk of serious adverse events
Mansouri et al [20]	2018	Yes	MEDLINE, EMBASE, CENTRAL, ISI Web	13	508	No	Yes	Yes	Yes	Yes	No	Deep brain stimulation of STN and GPi showed similar effect in reducing motor symptoms
Xie et al [21]	2016	Yes	PubMed, Google scholar, CENTRAL	16	2186	No	Yes	No	No	Yes	No	Both deep brain stimulation of GPi and STN is effective compared to best medical treatment
Elgebaly et al [22]	2018	No (only RCTs in meta-analysis)	PubMed, CENTRAL, ISI Web	7 (4 RCTs)	555 (345 from RCTs)	No	Yes	No	No	Yes	No	Deep brain stimulation of STN and GPi showed equal neuropsychological outcomes
*Essential tremor*												
Lu et al [24]	2020	No	PubMed, Embase, Cochrane Library	46	1714	Registry	Yes	Yes	No	Only publication bias	No	Deep brain stimulation is an effective and safe intervention but with a risk of adverse events
Altinel [23]	2019	Yes	PubMed, EMBASE, CENTRAL	15	1508	Registry	Yes	No	No	Yes	No	Lesion surgery and deep brain stimulation are equally effective in tremor reduction
*Dystonia*												
Rodrigues et al [25]	2019	Yes	CENTRAL, MEDLINE, EMBASE, Web of Science, SciELO, LILACS	2	102	Yes	Yes	Yes	Yes	Yes	Yes	Deep brain stimulation improves symptoms but there is uncertainty about the improvement of quality of life and safety
*Epilepsy*												
Sprengers et al [26]	2017	Yes	CENTRAL, Cochrane Epilepsy Group Specialized Register, PubMed, Clinicaltrials.gov, ICTRP	12	367	Yes	Yes	Yes	Yes	Yes	No	Deep brain stimulation reduced seizure frequency moderately depending on the placement of electrodes

*CENTRAL* Cochrane Central Register of Controlled Trials, *EMBASE* Excerpta Medica Database, *GPi* globus pallidus internus, *GRADE* Grading of Recommendations, Assessment, Development and Evaluation, *ICTRP* International Clinical Trials Registry Platform, *ISI Web* Web of Science, *LILACS* Latin American and Caribbean Health Sciences Literature, *MEDLINE* Medical Literature Analysis and Retrieval System Online, *RCT* randomised clinical trial, *SciELO* Scientific Electronic Library Online, *STN* subthalamic nucleus, *TSA* trial sequential analysis

In addition to our critical points, we have identified several relevant randomised clinical trials, which were not included in the abovementioned reviews [27–34]. Moreover, no systematic review has yet assessed deep brain stimulation as intervention for all neurological disorders. With an increasing use of deep brain stimulation as intervention for neurological disorders, there is a need for a comprehensive overview of the current evidence assessing both benefits and harms based on randomised clinical trials.

## Methods

The present protocol has been registered in the PROSPERO database (PROSPERO ID number: 306556) and is reported in accordance with the guidelines provided in Preferred Reporting Items for Systematic Reviews and Meta-Analysis Protocols (PRISMA-P) statement [35, 36] (see checklist in Additional file 1).

### Criteria for considering studies for this review

#### Types of studies

We will include randomised clinical trials irrespective of publication year, status, and language. We will include cross-over trials using only data from the first period of the trial. We will not include quasi-randomised trials, cluster-randomised trials, or non-randomised studies. If the reporting of methodology is limited resulting in doubt whether a trial is quasi-randomised or not, this will be addressed in risk of bias assessments and the interpretation of results.

#### Types of participants

Participants in all age groups with the diagnosis of either Parkinson’s disease, essential tremor, dystonia, or epilepsy will be included. As for the definition of Parkinson’s disease, essential tremor, dystonia, and epilepsy, we will accept the trialists’ definition. Participants will be included irrespective of sex, comorbidities, and risk factors.

#### Types of interventions

##### Experimental group

We will include any type of deep brain stimulation as intervention (as defined by trialists) independent of target of the electrodes, stimulation settings, unilateral or bilateral, and device used.

##### Control group

As control interventions, we will accept no intervention, usual care, sham stimulation, medical treatment, or resective surgery. The results of the different comparisons will be reported separately.

##### Cointerventions

We will accept any cointerventions, if these are planned to be delivered similarly in the experimental and control groups.

### Outcomes

#### Primary outcomes

1. All-cause mortality.
2. Disease-specific symptoms (continuous outcome). For each specific disease, we will accept any valid continuous scale assessing symptoms as defined by trialist (e.g. Unified Parkinson’s Disease Rating Scale for Parkinson’s disease, The Essential Tremor Rating Assessment Scale for essential tremor, The Burke-Fahn-Marsden Dystonia Rating Scale for dystonia, and The Liverpool Seizure Severity Scale for epilepsy).
3. The proportion of participants with one or more serious adverse events. We will use the International Conference on Harmonization of technical requirements for registration of pharmaceuticals for human use-Good Clinical Practice (ICH-GCP) definition of a serious adverse event, which is any untoward medical occurrence that resulted in death, was life-threatening, required hospitalisation or prolonging of existing hospitalisation, and resulted in persistent or significant disability or jeopardised the participant [37]. If the trialists do not use the ICH-GCP definition, we will include the data if the trialists use the term “serious adverse event”. If the trialists do not use the ICH-GCP definition nor use the term serious adverse event, we will then include the data if the event clearly fulfils the ICH-GCP definition for a serious adverse event.

#### Secondary outcomes

1. Quality of life assessed on any valid continuous scale
2. Depressive symptoms assessed on any valid continuous scale (e.g. Hamilton Depression Rating Scale)
3. Executive functioning measured on any valid scale (e.g. Wisconsin Card Sorting Test)
4. Level of functioning measured on any valid scale (e.g. Schwab and England Activities of Daily Living scale)
5. The proportion of participants with one or more non-serious adverse events (any adverse event not considered serious (see above))

#### Assessment time points

We will pragmatically use the trial results reported at maximum follow-up.

### Search methods for identification of studies

#### Electronic searches

We will search Cochrane Central Register of Controlled Trials (CENTRAL), Medical Literature Analysis and Retrieval System Online (MEDLINE), Excerpta Medica database (EMBASE), Latin American and Caribbean Health Sciences Literature (LI-LACS), Science Citation Index Expanded (SCI-EXPANDED), Conference Proceedings Citation Index— Science (CPCI-S), Chinese Biomedical Literature Database (CBM), China Network Knowledge Information (CNKI), Chinese Science Journal Database (VIP), and Wafang Database to identify relevant trials. We will search all databases from their inception to the present date. For a detailed search strategy for all electronic databases, see Additional file 2. We will include trials irrespective of language, publication year, publication status, and publication type.

#### Searching other resources

We will check the reference list of relevant trial publications for any unidentified clinical trials. We will contact the authors of included trials by email asking for unpublished randomised clinical trials. To identify unpublished trials, we will search clinical trial registries (e.g. clinicaltrials.gov), websites of US Food and Drug Administration (FDA), and European Medicines Agency (EMA). We will request FDA and EMA to provide all publicly releasable information about relevant randomised clinical trials of deep brain stimulation submitted for marketing approval. Furthermore, we will hand-search conference abstracts from neurosurgical conferences for relevant trials. We will search grey literature to include any unpublished and grey literature if we identify these and assess relevant retraction statements and errata for included trials.

### Data collection

We will perform and report the review as recommended by the Preferred Reporting Items for Systematic Reviews and Meta-Analyses (PRISMA) statement. Analyses will be performed using Stata (StataCorp LLC, College Station, TX, USA) [38] and Trial Sequential Analysis [39, 40].

#### Selection of randomised clinical trials

Two review authors will independently screen titles and abstracts. We will retrieve all relevant full-text study reports/publications, and two review authors will independently screen full-text and record reasons for excluding ineligible trials. Any disagreement will be solved by the same two authors by discussion, or if required, they will consult with a third author.

#### Data extraction and management

Two review authors will independently extract data from included trials in a predefined form. Disagreements will be solved by discussion with a third author. The two review authors will assess duplicate publications and companion papers of a trial together to evaluate all available data simultaneously (to maximise data extraction and correct bias assessment). Each trial will be named after the first author and year of the primary publication. We will contact the trial authors by email to specify any missing data, which may not have been reported sufficiently or at all in the publication.

#### Trial characteristics

We will extract the following data: bias risk components (as defined below), trial design (parallel, factorial, cross-over), number of intervention groups, length of follow-up, estimation of sample size, inclusion and exclusion criteria, for-profit funding of trial, and trial registration number.

#### Participant characteristics

We will extract the following data: number of randomised participants, number of analysed participants, number of participants lost to follow-up/withdrawals/crossovers, age range (mean or median), sex ratio, disease subtype, and disease severity at baseline.

#### Experimental intervention characteristics

We will extract the following data: full description of intervention including placement of electrodes, output voltage and current, stimulation frequency, pulse width, continuous, intermittent or responsive/closed-loop stimulation, device providers, duration of intervention, and co-interventions, if any.

#### Control intervention characteristics

We will extract the following: type of control intervention, duration of control intervention, co-interventions, if any. If the control intervention is best medical treatment, we will extract dose of control intervention. If control intervention is sham stimulation, we will extract placement of electrodes and device providers. If the control is resective surgery, we will extract characteristics of the surgical procedure.

#### Outcomes

We will extract all outcomes listed above from each randomised clinical trial. For each outcome, we will identify if outcomes are missing, inappropriately measured, or selectively reported according to the criteria described later in the “missing outcome data” bias domain, the “risk of bias in measurement of the outcome” bias domain, and the “risk of bias in selection of the reported results” bias domain.

#### Notes

We will search for information regarding industry funding of either personal or academic activities for each trial author. We will note in the “Characteristics of included studies” table if outcome data were not reported in a usable way. Disagreements will be solved by discussion, or, if required, we will consult with a third author.

### Assessment of risk of bias in the included studies

Our bias risk assessment will be based on the Cochrane Risk of Bias tool—version 2 (RoB 2) as recommended in The Cochrane Handbook of Systematic Reviews of Interventions [41]. Moreover, we will include an assessment of for-profit bias [42]. We will judge a publication at high risk of vested interests if a trial is sponsored by the industry or if just one author has affiliation to the industry. Additionally, we will evaluate the methodology in respect of the following bias domains:

#### Bias arising from the randomisation process

This domain encompasses allocation sequence generation and concealment as well as baseline differences between the trial arms.

##### Low risk of bias

Allocation was adequately concealed, AND imbalances across intervention groups at baseline appear to be compatible with chance, AND an adequate (random or otherwise unpredictable) method was used to generate allocation sequence, OR there is no information about the method used to generate the allocation sequence.

##### Some concerns

Allocation was adequately concealed, AND there is a problem with the method of sequence generation, OR baseline imbalances suggest a problem with the randomisation process, OR no information is provided about concealment of allocation, AND baseline imbalances across intervention groups appear to be compatible with chance, OR no information to answer any of the signalling questions.

##### High risk of bias

Allocation sequence was not concealed, OR no information is provided about concealment of allocation sequence, AND baseline imbalances suggest a problem with the randomisation process.

#### Bias due to deviation from intended interventions

##### Low risk of bias

Participants, carers, and personnel were unaware of intervention groups during the trial, OR participants, carers, or personnel were aware of intervention groups during the trial but any deviations from intended intervention reflected usual practice, OR participants, carers, or personnel were aware of intervention groups during the trial but any deviations from intended intervention were unlikely to impact on the outcome, AND no participants were analysed in the wrong intervention groups (that is, on the basis of intervention actually received rather than of randomised allocation).

##### Some concerns

Participants, carers, or personnel were aware of intervention groups and there is no information on whether there were deviations from usual practice that were likely to impact on the outcome and were imbalanced between intervention groups, OR some participants were analysed in the wrong intervention groups (based on intervention actually received rather than of randomised allocation) but there was little potential for a substantial impact on the estimated effect of intervention.

##### High risk of bias

Participants, carers, or personnel were aware of intervention groups, and there were deviations from intended interventions that were unbalanced between the intervention groups and likely to have affected the outcome, OR some participants were analysed in the wrong intervention groups (on the basis of intervention actually received rather than of randomised allocation), and there was potential for a substantial impact on the estimated effect of intervention.

#### Bias due to missing outcome data

##### Low risk of bias

No missing data OR non-differential missing data (similar proportion of and similar reasons for missing data in compared groups) OR evidence of robustness of effect estimate to missing data (based on adequate statistical methods for handling missing data and sensitivity analysis).

##### Some concerns

An unclear degree of missing data or unclear information on proportion and reasons for missingness in compared groups AND there is no evidence that the effect estimate is robust to missing data.

##### High risk of bias

A high degree of missing data AND differential missing data (different proportion of or different reasons for missing data in compared groups) AND there is no evidence that the effect estimate is robust to missing data.

#### Bias in measurement of the outcome

##### Low risk of bias

The outcome assessors were unaware of the intervention received by study participants, OR the outcome assessors were aware of the intervention received by study participants, but the assessment of the outcome was unlikely to be influenced by knowledge of the intervention received.

##### Some concerns

There is no information available to determine whether the assessment of the outcome is likely to be influenced by knowledge of the intervention received.

##### High risk of bias

The assessment of the outcome was likely to be influenced by knowledge of the intervention received by study participants.

#### Bias arising from selective reporting of results

##### Low risk of bias

Reported outcome data are unlikely to have been selected, on the basis of the results, from multiple outcome measurements (e.g. scales, definitions, time points) within the outcome domain, and reported outcome data are unlikely to have been selected, on the basis of the results, from multiple analyses of the data.

##### Some concerns

There is insufficient information available to exclude the possibility that reported outcome data were selected, on the basis of the results, from multiple outcome measurements (e.g. scales, definitions, time points) within the outcome domain, or from multiple analyses of the data. Given that analysis intentions are often unavailable or not reported with sufficient detail, we anticipate that this will be the default judgement for most trials.

##### High risk of bias

Reported outcome data are likely to have been selected, on the basis of the results, from multiple outcome measurements (e.g. scales, definitions, time points) within the outcome domain, or from multiple analyses of the data (or both).

#### Overall assessment of risk of bias

##### Low risk of bias

The study is judged to be at low risk of bias for all domains for this result.

##### High risk of bias

The study is judged to be at high risk of bias or to be at some concerns in at least one domain for this result. Our subgroup analysis will compare the intervention effect of trials at low risk of bias with trials at high risk of bias, that is one or more domains at some concerns or high risk of bias.

We will assess the domains “missing outcome data”, “risk of bias in measurement of the outcome”, and “risk of bias in selection of the reported result” for each outcome result. Thus, we can assess the bias risk for each outcome assessed in addition to each trial. Our primary conclusions will be based on the results of our primary outcome results with an overall low risk of bias. Both our primary and secondary conclusions will be presented in the summary of findings tables.

### Differences between the protocol and the review

We will conduct the review according to this published protocol. Any deviations from this will be described in the “Differences between the protocol and the review” section of the systematic review.

### Measurement of treatment effect

#### Dichotomous outcome

We will calculate risk ratios (RRs) with 95% confidence interval (CI) and Trial Sequential Analysis-adjusted CI for dichotomous outcomes.

#### Continuous outcomes

We will calculate the mean differences (MDs) with 95% CI and Trial Sequential Analysis-adjusted CI for continuous outcomes. If comparable continuous outcomes are reported, we will consider calculating the standardised mean difference (SMD) with 95% CI.

### Dealing with missing data

We will use intention-to-treat data if provided by the trialists [43]. We will, as the first option, contact all trial authors to obtain any relevant missing data (i.e. for data extraction and for assessment of risk of bias, as specified above), when individual patient data is not available.

#### Dichotomous outcome

We will not impute missing values for any outcomes in our primary analysis. In our sensitivity analyses (see paragraph below), we will impute data.

#### Continuous outcomes

We will primarily analyse scores assessed at maximum follow-up. If only changes from baseline scores are reported, we will analyse the results together with follow-up scores [41]. If standard deviations (SDs) are not reported, we will calculate the SDs using relevant trial data (e.g. *P* values), if possible. We will not use intention-to-treat data if the original report did not contain such data, per protocol data will then be used. In our best–worst-case and worst-best-case scenarios (see paragraph below) for continuous outcomes, we will impute data.

### Assessment of heterogeneity

We will primarily investigate forest plots to visually assess any sign of heterogeneity. We will secondly assess the presence of statistical heterogeneity using *I*^2^ statistic [41, 44, 45]*.* We will investigate evident heterogeneity through subgroup analyses (see Subgroup analyses and integration of heterogeneity section below). We may ultimately decide that a meta-analysis should be avoided if heterogeneity is significant [41].

### Assessment of reporting biases

We will use a funnel plot to assess reporting bias if ten or more trials are included [41]. We will visually inspect funnel plots to assess the risk of bias. We are aware of the limitations of a funnel plot (i.e. a funnel plot assesses bias due to small sample size) [41]. From this information, we will assess possible reporting bias. For dichotomous outcomes, we will test asymmetry with the Harbord test [46] if *τ*^2^ is less than 0.1 and with the Rücker test if *τ*^2^ is more than 0.1 [41]. For continuous outcomes, we will use the regression asymmetry test [47] and the adjusted rank correlation [48].

### Unit of analysis issues

We will only include randomised clinical trials. For trials using crossover design, only data from the first period will be included [41, 49]. There will therefore not be any unit of analysis issues. We will not include cluster-randomised trials.

### Data synthesis

#### Meta-analysis

We will undertake the meta-analysis according to the Cochrane Handbook for Systematic Reviews of Interventions [41], Keus et al. [50], and the eight-step procedure suggested by Jakobsen et al. [51]. We will use the statistical software Stata to analyse data (command: meta) [38]. We will assess the intervention effects with both random-effects meta-analyses [52] and fixed-effect meta-analyses for each comparison separately [53]. We will primarily use the most conservative result (highest *P* value) of the two and the least conservative result as a sensitivity analysis [51]. We will assess a total of three primary outcomes, and we will therefore consider a *P* value < 0.025 as the threshold for statistical significance for the primary outcomes [51]. All other outcomes will be considered hypothesis-generating only, and we will therefore use a threshold of *P* < 0.05 for all remaining outcomes.

We will separately assess the effects of deep brain stimulation as intervention for Parkinson’s disease, essential tremor, dystonia, and epilepsy. We will present these results and analyses in different sections of the same review, and in separate meta-analysis and Trial Sequential Analysis, we will analyse the results according to different control interventions. If we identify trials including participants with more than one neurological disorder, these will be included in the analyses for both disorders. However, we will make it very clear if this is the case. If we identify a large number of trials, we may consider making separate reviews for each neurological disorder. In a separate future review, we will conduct analyses of all diseases combined and avoid double counting.

We will investigate possible heterogeneity through subgroup analyses. We will use our eight-step procedure to assess if the thresholds for significance are crossed [51]. This eight-step procedure is comprised of the following steps: (1) obtain the 95% confidence intervals and the *P* values from both fixed-effect and random-effects meta-analyses and report the most conservative results as the main results; (2) explore the reasons behind substantial statistical heterogeneity using subgroup and sensitivity analyses (see step 6); (3) adjust the thresholds for significance according to the number of primary outcomes to take account of problems with multiplicity; (4) calculate required information sizes (≈ the a priori required number of participants for a meta-analysis to be conclusive) for all outcomes and analyse each outcome with Trial Sequential Analysis. Report whether the trial sequential monitoring boundaries for benefit, harm, or futility are crossed; (5) calculate Bayes factors for all primary outcomes; (6) use subgroup analyses and sensitivity analyses to assess the potential impact of bias on the review results; (7) assess the risk of publication bias; (8) assess the clinical significance of the statistically significant review results [51].

#### Trial Sequential Analysis

Traditional meta-analysis runs the risk of random errors due to sparse data and repetitive testing of accumulating data when updating reviews [54]. We wish to control the risks of both type I errors and type II errors [54]. We will therefore perform Trial Sequential Analysis on all outcomes, in order to calculate the required information size (that is, the number of participants needed in a meta-analysis to detect or reject a certain intervention effect) and the cumulative *Z*-curve’s breach of relevant trial sequential monitoring boundaries [39, 40, 55–61]. A more detailed description of Trial Sequential Analysis can be found in the Trial Sequential Analysis manual [40] and at http://www.ctu.dk/tsa/. For dichotomous outcomes, we will estimate the required information size based on the observed proportion of patients with an outcome in the control group (the cumulative proportion of patients with an event in the control groups relative to all patients in the control groups), a relative risk reduction of 10%, an alpha of 2.5% for the primary outcomes and 5% for the remaining outcomes, a beta of 10%, and the observed diversity as suggested by the trials in the meta-analysis. For continuous outcomes, we will in the Trial Sequential Analysis use the observed standard deviation (SD), a mean difference equal to the observed SD/2, an alpha of 2.5% for the primary outcomes and 5% for the remaining outcomes, a beta of 10%, and the observed diversity as suggested by the trials in the meta-analysis.

### Subgroup analyses and integration of heterogeneity

#### Subgroup analyses

We will perform the following subgroup analyses when analysing the primary outcomes (all-cause mortality, disease-specific symptoms, and serious adverse events).

- Trials at high risk of bias compared to trials at low risk of bias
- Trials without vested interests compared to trials with unknown or known risk of vested interests [42]
- Trials published before 2000 compared to trials published after 2000
- Target nucleus (e.g. subthalamic nucleus, internal globus pallidus, ventral intermedius nucleus)
- Types of comparators (e.g. no intervention, usual care, sham stimulation, medical treatment, or resective surgery)
- Disease subtypes (e.g. generalised epilepsy, focal epilepsy)
- Disease severity

We will use the formal test for subgroup interactions in Stata [38].

#### Sensitivity analysis

To assess the potential impact of the missing data for dichotomous outcomes, we will perform the two following sensitivity analyses on all primary and secondary dichotomous outcomes.

- Best–worst-case scenario: We will assume that all participants lost to follow-up in the experimental group survived and had no serious and non-serious adverse events and that all those participants lost to follow-up in the control group did not survive and had a serious and non-serious adverse event.
- Worst-best-case scenario: We will assume that all participants lost to follow-up in the experimental group did not survive and had a serious and non-serious adverse event and that all those participants lost to follow-up in the control group survived and had no serious and non-serious adverse event.

We will present results of both scenarios in our review. When analysing disease-specific symptoms and quality of life, a beneficial outcome will be the group mean plus two SDs of the group mean, and a harmful outcome will be the group mean minus two SDs of the group mean [51]. To assess the potential impact of missing SDs for continuous outcomes, we will perform the following sensitivity analysis:

- Where SDs are missing and it is not possible to calculate them, we will impute SDs from trials with similar populations and low risk of bias. If we find no such trials, we will impute SDs from trials with a similar population. As the final option, we will impute the mean SD from all included trials.

We will present results of this scenario in our review. Other post hoc sensitivity analyses might be warranted if unexpected clinical or statistical heterogeneity is identified during the analysis of the review results [51].

### Summary of findings table

We will create a summary of findings table for each comparison (deep brain stimulation vs. no intervention, usual care, sham stimulation, medical treatment, or resective surgery) including each of the prespecified outcomes (all-cause mortality, disease-specific symptoms, serious adverse events, quality of life, depressive symptoms, executive functioning, level of functioning, and non-serious adverse events). We will use the Grading of Recommendations, Assessment, Development and Evaluations (GRADE) considerations (bias risk of the trials, consistency of effect, imprecision, indirectness, and publication bias) to assess the certainty of evidence [51, 62–64]. We will assess imprecision using Trial Sequential Analysis. We will downgrade imprecision in GRADE by two levels if the accrued number of participants is below 50% of the diversity-adjusted required information size (DARIS), and one level if between 50 and 100% of DARIS. We will not downgrade if the cumulative *Z*-curve crosses the monitoring boundaries for benefit, harm, or futility, or DARIS is reached. We will justify all decisions to downgrade the certainty of evidence using footnotes, and we will make comments to aid the reader’s understanding of the review where necessary. Firstly, we will present our results in the summary of findings table based on the results from the trials with an overall low risk of bias, and secondly, we will present the results based on all trials.

## Discussion

This systematic review with meta-analyses and Trial Sequential Analysis of randomised clinical trials aims at assessing the beneficial and harmful effects of deep brain stimulation versus no intervention, usual care, sham stimulation, medical treatment, or resective surgery for participants with Parkinson’s disease, essential tremor, dystonia, or epilepsy and the neurological disorders combined. Primary outcomes will be all-cause mortality, disease-specific symptoms, and the proportion of participants with one or more serious adverse events. Secondary outcomes will be quality of life, depressive symptoms, executive functioning, level of functioning, and non-serious adverse events.

One of the strengths of our protocol is the methodological approach. The predefined methodology is based on Keus et al. [50], our eight-step assessment suggested by Jakobsen et al. [51], Trial Sequential Analysis [39], and GRADE assessment of the certainty of evidence [62–64]. Therefore, we consider both the risk of random errors and the risk of systematic errors. Furthermore, we will include data from published trials, unpublished trials, and clinical study reports and thereby reduce the risk of publication bias.

Another strength of this protocol is the intention to assess deep brain stimulation for different neurological disorders altogether. This allows for an individual assessment of the intervention related to the specific neurological disorder as well as an overview of the intervention related to all the selected neurological disorders.

Our protocol also has some limitations. The primary limitation is the risk of identifying a limited number of randomised clinical trials. Furthermore, there is a risk of high statistical heterogeneity among the included trials. This will be addressed in the analyses for heterogeneity, subgroup analyses, and sensitivity analyses.

Another limitation is the possibility of many comparisons due to different kinds of deep brain stimulation and control interventions. This increases the risk of type I errors. Although we have adjusted the threshold for significance according to the number of outcomes, the threshold for significance has not been adjusted according to the number of different diseases, number of different control interventions, or subgroup analyses.

Lastly, we have decided to only include randomised clinical trials. By excluding cluster-randomised trials, quasi-randomised trials, and observational studies, there is a risk of overlooking rare and late-occurring adverse events. If we do find a beneficial effect of deep brain stimulation compared with no intervention, sham stimulation, medical treatment, or resective surgery, it will be relevant to assess rare and late-occurring adverse events according to cluster-randomised trials, quasi-randomised trials, and observational studies.

## Supplementary Information

**Additional file 1. **PRISMA-P (Preferred Reporting Items for Systematic review and Meta-Analysis Protocols) 2015 checklist.

**Additional file 2. **Search strategy for all electronic databases.

## Data Availability

Not applicable.

## Abbreviations

CBM: Chinese Biomedical Literature Database
CENTRAL: Cochrane Central Register of Controlled Trials
CI: Confidence interval
CNKI: China Network Knowledge Information
CPCI-S: Conference Proceedings Citation Index—Science
DARIS: Diversity-adjusted required information size
EMA: European Medicines Agency
EMBASE: Excerpta Medica database
FDA: US Food and Drug Administration
GRADE: The Grading of Recommendations Assessment, Development and Evaluation
ICH-GCP: International Conference on Harmonization of technical requirements for registration of pharmaceuticals for human use—Good Clinical Practice
LI-LACS: Latin American and Caribbean Health Sciences Literature
MD: Mean differences
MEDLINE: Medical Literature Analysis and Retrieval System Online
PRISMA: Preferred reporting items for systematic review and meta-analysis
PRISMA-P: Preferred reporting items for systematic review and meta-analysis—protocols
PROSPERO: International Prospective Register of Systematic Reviews
RR: Risk ratio
SCI-EXPANDED: Science index citation expanded
SD: Standard deviation
SMD: Standardised mean difference
VIP: Chinese Science Journal Database
WHO: World Health Organization

## References

[1] Feigin VL, Nichols E, Alam T, Bannick MS, Beghi E, Blake N (2019). Global, regional, and national burden of neurological disorders, 1990–2016: a systematic analysis for the Global Burden of Disease Study 2016. The Lancet Neurology.

[2] Freitas ME, Ruiz-Lopez M, Dalmau J, Erro R, Privitera M, Andrade D (2019). Seizures and movement disorders: phenomenology, diagnostic challenges and therapeutic approaches. J Neurol Neurosurg Psychiatry.

[3] Hayes MW, Fung VS, Kimber TE, O’Sullivan JD (2010). Current concepts in the management of Parkinson’s disease. Med J Aust.

[4] Bloem BR, Okun MS, Klein C (2021). Parkinson's disease. Lancet.

[5] Fasano A, Daniele A, Albanese A (2012). Treatment of motor and non-motor features of Parkinson's disease with deep brain stimulation. Lancet Neurology.

[6] Haubenberger D, Hallett M (2018). Essential tremor. N Engl J Med.

[7] Wong JK, Hess CW, Almeida L, Middlebrooks EH, Christou EA, Patrick EE (2020). Deep brain stimulation in essential tremor: targets, technology, and a comprehensive review of clinical outcomes. Expert Rev Neurother.

[8] Phukan J, Albanese A, Gasser T, Warner T (2011). Primary dystonia and dystonia-plus syndromes: clinical characteristics, diagnosis, and pathogenesis. Lancet Neurology.

[9] Albanese A, Bhatia K, Bressman SB, Delong MR, Fahn S, Fung VS (2013). Phenomenology and classification of dystonia: a consensus update. Mov Disord.

[10] Termsarasab P, Thammongkolchai T, Frucht SJ (2016). Medical treatment of dystonia. J Clin Mov Disord.

[11] Simonato M (2018). Epilepsy an update on disease mechanisms: the potential role of MicroRNAs. Front Neurol.

[12] Forsgren L, Beghi E, Õun A, Sillanpää M (2005). The epidemiology of epilepsy in Europe – a systematic review. Eur J Neurol.

[13] Wu YC, Liao YS, Yeh WH, Liang SF, Shaw FZ (2021). Directions of deep brain stimulation for epilepsy and Parkinson's disease. Front Neurosci.

[14] Hanaya R, Arita K (2016). The new antiepileptic drugs: their neuropharmacology and clinical indications. Neurol Med Chir (Tokyo).

[15] Lozano AM, Lipsman N, Bergman H, Brown P, Chabardes S, Chang JW (2019). Deep brain stimulation: current challenges and future directions. Nat Rev Neurol.

[16] Aum DJ, Tierney TS (2018). Deep brain stimulation: foundations and future trends. Front Biosci.

[17] Boon P, Raedt R, de Herdt V, Wyckhuys T, Vonck K (2009). Electrical stimulation for the treatment of epilepsy. Neurotherapeutics..

[18] Wong JK, Cauraugh JH, Ho KWD, Broderick M, Ramirez-Zamora A, Almeida L (2019). STN vs. GPi deep brain stimulation for tremor suppression in Parkinson disease: a systematic review and meta-analysis. Parkinsonism Relat Disord..

[19] Bratsos S, Karponis D, Saleh SN (2018). Efficacy and safety of deep brain stimulation in the treatment of Parkinson's disease: a systematic review and meta-analysis of randomized controlled trials. Cureus.

[20] Mansouri A, Taslimi S, Badhiwala JH, Witiw CD, Nassiri F, Odekerken VJJ (2018). Deep brain stimulation for Parkinson's disease: meta-analysis of results of randomized trials at varying lengths of follow-up. J Neurosurg.

[21] Xie CL, Shao B, Chen J, Zhou Y, Lin SY, Wang WW (2016). Effects of neurostimulation for advanced Parkinson's disease patients on motor symptoms: a multiple-treatments meta-analysas of randomized controlled trials. Sci Rep.

[22] Elgebaly A, Elfil M, Attia A, Magdy M, Negida A (2018). Neuropsychological performance changes following subthalamic versus pallidal deep brain stimulation in Parkinson's disease: a systematic review and metaanalysis. CNS Spectr.

[23] Altinel Y, Alkhalfan F, Qiao N, Velimirovic M (2019). Outcomes in lesion surgery versus deep brain stimulation in patients with tremor: a systematic review and meta-analysis. World Neurosurg.

[24] Lu G, Luo L, Liu M, Zheng Z, Zhang B, Chen X (2020). Outcomes and adverse effects of deep brain stimulation on the ventral intermediate nucleus in patients with essential tremor. Neural Plast.

[25] Rodrigues FB, Duarte GS, Prescott D, Ferreira J, Costa J (2019). Deep brain stimulation for dystonia. Cochrane Database Syst Rev..

[26] Sprengers M, Vonck K, Carrette E, Marson AG, Boon P (2017). Deep brain and cortical stimulation for epilepsy. Cochrane Database Syst Rev..

[27] Troster AI, Jankovic J, Tagliati M, Peichel D, Okun MS (2017). Neuropsychological outcomes from constant current deep brain stimulation for Parkinson's disease. Mov Disord.

[28] Hacker ML, Turchan M, Heusinkveld LE, Currie AD, Millan SH, Molinari AL (2020). Deep brain stimulation in early-stage Parkinson’s disease: five-year outcomes. Neurology.

[29] Esselink RAJ, de Bie RMA, de Haan RJ, Lenders MWPM, Nijssen PCG, Staal MJ (2004). Unilateral pallidotomy versus bilateral subthalamic nucleus stimulation in PD. Neurology.

[30] Schuurman PR, Bosch DA, Bossuyt PM, Bonsel GJ, van Someren EJ, de Bie RM (2000). A comparison of continuous thalamic stimulation and thalamotomy for suppression of severe tremor. N Engl J Med.

[31] Anderson VC, Burchiel KJ, Hart MJ, Berk C, Lou JS (2009). A randomized comparison of thalamic stimulation and lesion on self-paced finger movement in essential tremor. Neurosci Lett.

[32] Gruber D, Sudmeyer M, Deuschl G, Falk D, Krauss JK, Mueller J (2018). Neurostimulation in tardive dystonia/dyskinesia: A delayed start, sham stimulation-controlled randomized trial. Brain Stimul.

[33] Cukiert A, Cukiert CM, Burattini JA, Mariani PP, Bezerra DF (2017). Seizure outcome after hippocampal deep brain stimulation in patients with refractory temporal lobe epilepsy: a prospective, controlled, randomized, double-blind study. Epilepsia.

[34] Herrman H, Egge A, Konglund AE, Ramm-Pettersen J, Dietrichs E, Tauboll E (2019). Anterior thalamic deep brain stimulation in refractory epilepsy: a randomized, double-blinded study. Acta Neurol Scand.

[35] Moher D, Shamseer L, Clarke M, Ghersi D, Liberati A, Petticrew M (2015). Preferred reporting items for systematic review and meta-analysis protocols (PRISMA-P) 2015 statement. BioMed Central.

[36] Shamseer L, Moher D, Clarke M, Ghersi D, Liberati A, Petticrew M (2015). Preferred reporting items for systematic review and meta-analysis protocols (PRISMA-P) 2015: elaboration and explanation. BMJ.

[37] ICH harmonised guideline: Integrated addemdum to ICH E6(R1): Guideline for Good Clinical Practice (ICH-GCP). International Conference on Harmonisation of Technical Requirements for Registration of Pharmaceuticals for Human Use; 2015. Available from: https://ichgcp.net/da.

[38] StataCorp. Stata Statistical Software: Release 16 2019. College Station, TX: StataCorp LLC http://www.stata.com.

[39] Thorlund K, Engstrøm J, Wetterslev J, Brok J, Imberger G, Gluud C. Copenhagen Trial Unit. TSA - trial sequential analysis. Available from: http://www.ctu.dk/tsa/.

[40] Thorlund K, Engstrøm JW, Jørn., Brok J, Imberger G, Gluud C. User Manual for Trial Sequential Analysis (TSA) Copenhagen, Denmark: Copenhagen Trial Unit, Centre for Clinical Intervention Research. Available from: http://www.ctu.dk/tsa/files/tsa_manual.pdf.

[41] Higgins J, Thomas J, Chandler J, Cumpston M, Li T, Page M, et al. Cochrane Handbook for Systematic Reviews of Interventions version 6.2 (updated February 2021) Cochrane: Cochrane; 2021 [Available from: www.training.cochrane.org/handbook.

[42] Lundh A, Lexchin J, Mintzes B, Schroll JB, Bero L (2018). Industry sponsorship and research outcome: systematic review with meta-analysis. Intensive Care Med.

[43] Jakobsen JC, Gluud C, Wetterslev J, Winkel P (2017). When and how should multiple imputation be used for handling missing data in randomised clinical trials - a practical guide with flowcharts. BMC Med Res Methodol.

[44] Higgins JP, Thompson SG (2002). Quantifying heterogeneity in a meta-analysis. Stat Med.

[45] Higgins JP, Thompson SG, Deeks JJ, Altman DG (2003). Measuring inconsistency in meta analyses. BMJ.

[46] Harbord RM, Egger M, Sterne JA (2006). A modified test for small-study effects in meta-analyses of controlled trials with binary endpoints. Stat Med.

[47] Egger M, George DS, Schneider M, Minder C (1997). Bias in meta-analysis detected by a simple, graphical test. BMJ.

[48] Begg C, Mazumdar M (1994). Operating characteristics of a rank correlation test for publication bias. Int Biom Soc.

[49] Elbourne DR, Altman DG, Higgins JP, Curtin F, Worthington HV, Vail A (2002). Meta-analyses involving cross-over trials: methodological issues. Int J Epidemiol.

[50] Keus F, Wetterslev J, Gluud C, van Laarhoven CJ (2010). Evidence at a glance: error matrix approach for overviewing available evidence. BMC Med Res Methodol.

[51] Jakobsen JC, Wetterslev J, Winkel P, Lange T, Gluud C (2014). Thresholds for statistical and clinical significance in systematic reviews with meta-analytic methods. BioMed Central.

[52] DerSimonian R, Laird N (1986). Meta-analysis in clinical trials. Elsevier Science Publishing.

[53] DeMets D (1987). Methods for combining randomized clinical trials: strengths and limitations. Stat Med.

[54] Wetterslev J, Jakobsen JC, Gluud C (2017). Trial Sequential Analysis in systematic reviews with meta-analysis. BMC Med Res Methodol.

[55] Wetterslev J, Thorlund K, Brok J, Gluud C (2008). Trial sequential analysis may establish when firm evidence is reached in cumulative meta-analysis. J Clin Epidemiol.

[56] Brok J, Thorlund K, Gluud C, Wetterslev J (2008). Trial sequential analysis reveals insufficient information size and potentially false positive results in many meta-analyses. J Clin Epidemiol.

[57] Brok J, Thorlund K, Wetterslev J, Gluud C (2009). Apparently conclusive meta-analyses may be inconclusive–Trial sequential analysis adjustment of random error risk due to repetitive testing of accumulating data in apparently conclusive neonatal meta-analyses. Int J Epidemiol.

[58] Thorlund K, Devereaux PJ, Wetterslev J, Guyatt G, Ioannidis JP, Thabane L (2009). Can trial sequential monitoring boundaries reduce spurious inferences from meta-analyses?. Int J Epidemiol.

[59] Wetterslev J, Thorlund K, Brok J, Gluud C (2009). Estimating required information size by quantifying diversity in random-effects model meta-analyses. BMC Med Res Methodol.

[60] Horlund K, Anema A, Mills E (2010). Interpreting meta-analysis according to the adequacy of sample size. An example using isoniazid chemoprophylaxis for tuberculosis in purified protein derivative negative HIV-infected individuals. Clinical Epidemiology..

[61] Imberger G, Thorlund K, Gluud C, Wetterslev J (2016). False-positive findings in Cochrane meta-analyses with and without application of trial sequential analysis: an empirical review. BMJ Open.

[62] Guyatt GH, Oxman AD, Vist GE, Kunz R, Falck-Ytter Y, Alonso-Coello P (2008). GRADE: an emerging consensus on rating quality of evidence and strength of recommendations. BMJ.

[63] Guyatt GH, Oxman AD, Schunemann HJ, Tugwell P, Knottnerus A (2011). GRADE guidelines: a new series of articles in the Journal of Clinical Epidemiology. J Clin Epidemiol.

[64] Schünemann HJ, Best D, Vist G, Oxman AD (2003). Letters, numbers, symbols and words: how to communicate grades of evidence and recommendations. Can Med Assoc J.

