# Supplementary material for: Deep brain stimulation for neurological disorders: a protocol for a systematic review with meta-analysis and Trial Sequential Analysis of randomised clinical trials
Source: Syst Rev. 2022 Oct 13;11:218. doi: 10.1186/s13643-022-02095-z (PMC9558400; doi:10.1186/s13643-022-02095-z)
Supplement: Supplementary file 2 — Additional file 2. Search strategy for all electronic databases. [file 13643_2022_2095_MOESM2_ESM.doc]

# Search strategies for

# Deep brain stimulation for treatment of neurological diseases

# (J Juul Petersen)

# Preliminary searches prepared 17 January 2022

Cochrane Central Register of Controlled Trials (latest issue) in the Cochrane Library (*in 2022, Issue 1: 4804 hits*)

#1 MeSH descriptor: [Deep Brain Stimulation] explode all trees

#2 MeSH descriptor: [Electric Stimulation Therapy] this term only

#3 MeSH descriptor: [Thalamus] explode all trees

#4 ((((brain and (deep or depth)) or thala* or elective or neuro*) and (stimul* or excitation*)) or DBS)

#5 #1 or #2 or #3 or #4

#6 MeSH descriptor: [Parkinson Disease] explode all trees

#7 MeSH descriptor: [Tremor] explode all trees

#8 MeSH descriptor: [Essential Tremor] explode all trees

#9 MeSH descriptor: [Dystonia] explode all trees

#10 MeSH descriptor: [Epilepsy] explode all trees

#11 (parkinson* or PD or tremor or ET or dystoni* or epilep*)

#12 #6 or# 7 or# 8 or #9 or #10 or #11

#13 #5 and #12

**MEDLINE Ovid (1946 to the date of the search) (*on 17 January 2022: 2706 hits*)**

1. exp Deep Brain Stimulation/

2. Electric Stimulation Therapy/

3. exp Thalamus/

4. ((((brain and (deep or depth)) or thala* or elective or neuro*) and (stimul* or excitation*)) or DBS).mp. [mp=title, abstract, original title, name of substance word, subject heading word, floating sub-heading word, keyword heading word, organism supplementary concept word, protocol supplementary concept word, rare disease supplementary concept word, unique identifier, synonyms]

5. 1 or 2 or 3 or 4

6. exp Parkinson Disease/

7. exp Tremor/

8. exp Essential Tremor Essential Tremor /

9. exp Dystonia/

10. exp Epilepsy/

11. (parkinson* or PD or tremor or ET or dystoni* or epilep*).mp. [mp=title, abstract, original title, name of substance word, subject heading word, floating sub-heading word, keyword heading word, organism supplementary concept word, protocol supplementary concept word, rare disease supplementary concept word, unique identifier, synonyms]

12. 6 or 7 or 8 or 9 or 10 or 11

13. 5 and 12

14. (randomized controlled trial or controlled clinical trial or retracted publication or retraction of publication).pt. or clinical trials as topic.sh. or trial.ti.

15. (random* or blind* or placebo* or meta-analys*).mp.

16. 13 and (14 or 15)

17. exp animals/ not humans.sh.

18. 16 not 17

**Embase Ovid (1974 to the date of the search) (*on 17 January 2022: 14467 hits*)**

1. exp brain depth stimulation/

2. electrotherapy/

3. exp thalamus/

4. ((((brain and (deep or depth)) or thala* or elective or neuro*) and (stimul* or excitation*)) or DBS).mp. [mp=title, abstract, heading word, drug trade name, original title, device manufacturer, drug manufacturer, device trade name, keyword heading word, floating subheading word, candidate term word]

5. 1 or 2 or 3 or 4

6. exp Parkinson disease/

7. exp tremor/

8. exp dystonia/

9. exp epilepsy/

10. (parkinson* or PD or tremor or ET or dystoni* or epilep*).mp. [mp=title, abstract, heading word, drug trade name, original title, device manufacturer, drug manufacturer, device trade name, keyword heading word, floating subheading word, candidate term word]

11. 6 or 7 or 8 or 9 or 10

12. 5 and 11

13. Randomized controlled trial/ or Controlled clinical trial/ or retracted article/ or (erratum or tombstone).pt. or trial.ti. or yes.nr.

14. (random* or blind* or placebo* or meta-analys*).mp. [mp=title, abstract, heading word, drug trade name, original title, device manufacturer, drug manufacturer, device trade name, keyword heading word, floating subheading word, candidate term word]

15. 12 and (13 or 14)

16. Animal experiment/ not (human experiment/ or human/)

17. 15 not 16

**LILACS (Bireme; 1982 to the date of the search) (*on 17 January 2022: 640 hits*)**

((((brain and (deep or depth)) or thala$ or elective or neuro$) and (stimul$ or excitation$)) or DBS) [Words] and (parkinson$ or PD or tremor or ET or dystoni$ or epilep$) [Words]

**Science Citation Index Expanded (1900 to the date of the search) and** **Conference Proceedings Citation Index – Science (1990 to the date of the search) (Web of Science) (*on 17 January 2022: 3731 hits*)**

#5 #3 AND #4

#4 TI=(random* or blind* or placebo* or meta-analys* or trial*) OR TS=(random* or blind* or placebo* or meta-analys*)

#3 #2 AND #1

#2 TS=(parkinson* or PD or tremor or ET or dystoni* or epilep*)

#1 TS=((((brain and (deep or depth)) or thala* or elective or neuro*) and (stimul* or excitation*)) or DBS)
